# Does only thymectomy influence on results of surgical treatment at patients with myasthenia gravis?

**DOI:** 10.1186/1749-8090-8-S1-P181

**Published:** 2013-09-11

**Authors:** S Pushkin, A Benyan, A Reshetov

**Affiliations:** 1 Samara Regional Clinical Hospital, Samara, Russian Federation

## Background

Among the all autoimmune diseases myasthenia gravis has etiological and pathogenic causal relationship with pathology of thymus. Performing of thymectomy helps to treatment of patients with myasthenia gravis. Presence of thymus corpuscles in mediastinal fat occurs in 10-25%, so dissection of mediastinal fat can prevent possible recurrence of disease.

## Methods

We have analyzed results of surgical treatment of 87 patients having myasthenia gravis. There were 68 (79,2%) female, 19 (21,8%) male. Patients ranged in age from 15 to 64 years. 80 patients (91,9%) had generalized myasthenia, 7 patients (8,1%) had local forms of myasthenia. The most number of patients had IIb – Iva degree of myasthenia according to international clinical classification. All patients underwent extended thymectomy. At 75 patients we have performed thymectomy by median sternotomy, at 10 patients – by VATS. Dissection of mediastinal fat was a mandatory component of operation. The histologic pattern was: thymoma – at 43 patients (49,4%), thymic hyperplasia – at 42 patient (48,3%), thymic atrophy – at 2 patients (2,3%). Additional thymus corpuscles were revealed at 10 patients (11,5%).

## Results

The time of observing after operation was from 6 months to 9 years. Relapse of thymoma was registered at 1 patient (1,1%). For assessment of clinical results we used the modified Keynes`s scale. We have got perfect and good results (groups A and B) at 65 patients (74,7%). These results were observed at 34 patients (80,9%) with thymic hyperplasia and 31 patients (72,1%) with thymoma. Also, best results we have got at 71 patient (81,6%), whom operation had been performed in first 2 years after the onset of disease. Mortality was 4,6% (4 patients).

## Conclusions

Thymectomy is a part of complex treatment of patients with myasthenia. Performing of dissection of mediastinal fat is necessary. Young age of patient and short period before operation affect on results, too.

